# Risk assessment of prolonged jaundice in infants at one month of age: A prospective cohort study

**DOI:** 10.1038/s41598-018-33249-6

**Published:** 2018-10-04

**Authors:** Yi-Hao Weng, Shao-Wen Cheng, Chun-Yuh Yang, Ya-Wen Chiu

**Affiliations:** 1 Department of Pediatrics, Chang Gung Memorial Hospital, Chang Gung University College of Medicine, Taipei, Taiwan; 2 Department of Public Health, Kaohsiung Medical University, Kaohsiung, Taiwan; 3 Food and Drug Administration, Ministry of Health and Welfare, Taipei, Taiwan; 4 Master Program in Global Health and Development, College of Public Health, Taipei Medical University, Taipei, Taiwan

## Abstract

Prolonged jaundice is a commonly evaluated condition. The aim of this study was to assess the risk factors of jaundice in healthy infants at one month of age. This prospective cohort study enrolled 509 healthy infants from 2013 to 2018. Those with gestational age (GA) less than 35 weeks, birth weight less than 2000 grams, and illness were not enrolled. Jaundice was defined as a transcutaneous bilirubin value ≥5 mg/dL at 25–45 days of age. Umbilical cord blood samples were obtained to examine seven common gene variants. The incidence of prolonged jaundice was 32.2%. Prolonged jaundice was more common in infants with exclusive breastfeeding (p < 0.001), GA 35~37 w (p = 0.001), stool passage >4 times/d (p < 0.001), previous phototherapy (p < 0.001), and gene variant of G to A at nt 211 of UGT1A1 (p = 0.004). A multivariate logistic regression analysis demonstrated the greatest risk for prolonged jaundice was exclusive breastfeeding (OR = 2.818, 95% CI = 1.851–4.292), followed by previous phototherapy (OR = 2.593, 95% CI = 1.716–3.919), GA 35~37 w (OR = 2.468, 95% CI = 1.350–4.512), and G to A at nt 211 of UGT1A1 (OR = 1.645, 95% CI = 1.070–2.528). In conclusion, infants with exclusive breastfeeding, GA 35~37 w, previous phototherapy, or G to A at nt 211 of UGT1A1 are at greater risk of prolonged jaundice. Healthcare professionals should consider these risk factors in their assessment of prolonged jaundice.

## Introduction

Neonatal jaundice is associated with a variety of physiologic and pathologic conditions^1^. Gene variants involving the production and metabolism of bilirubin are risk factors of neonatal jaundice – including glucose-6-phosphate dehydrogenase (G6PD)^2^, blood group^3^, heme oxygenase (HO)−1^4^, hepatic solute carrier organic anion transporter 1B1 (SLCO1B1)^5^, and UDP-glucuronosyltransferase 1A1 (UGT1A1)^6,7^.

Prolonged jaundice, defined as visible jaundice beyond 14 days, is one of the most commonly evaluated conditions during neonatal and young infant period in Asian population^8^. It can be a sign of a serious underlying pathology. Nevertheless, the vast majority of prolonged jaundice cases are of benign origin. Breastfeeding has been the leading cause of prolonged jaundice worldwide^9^. The mechanism of breast milk jaundice is not clearly understood yet. A number of theories have arisen to explain it, such as genetic factors. Many studies have documented the strong association between breast milk jaundice and gene variants of TA repeat or nucleotide (nt) 211 in UGT1A1 promoter^8,10,11^. In addition, gene variants of GT repeat in HO-1 promoter have been reported as a risk factor for prolonged jaundice^12^.

To date, a large-scale comprehensive approach to investigate the risk of prolonged jaundice in healthy infants is lacking. In this prospective cohort survey, we examined the clinical manifestations and genetic variants of one-month-old infants to verify their correlation with prolonged jaundice.

## Methods

### Study design

This prospective study involved exploratory research conducted through examinations of infants and interviews with nursing parents. Healthy infants born in the Chang Gung Memorial Hospital at Taipei between January 2013 and May 2018 were eligible for enrollment. Those with gestational age (GA) less than 35 weeks, birth weight less than 2000 grams, and illness (such as significant congenital anomaly) were excluded. The Institutional Review Board of Chang Gung Memorial Hospital approved the study protocol. All experiments were performed in accordance with relevant guidelines and regulations. Informed consent was obtained from parents of enrolled infants.

### Clinical measures

Bilirubin level and concurrent body weight were examined for infants at 25 to 45 days of age in the well-baby clinics. Bilirubin was measured with non-invasive transcutaneous bilirubinmeter BiliCheck device (Spectrx Inc, Norcross, GA, USA). Jaundice was defined as a transcutaneous bilirubin (TcB) value ≥5 mg/dL. The devices for measuring the TcB value and body weight were the same through the whole study period.

Feeding type and stool pattern were obtained by interviews with parents. Feeding type was classified into three categories: (1) formula feeding; (2) combination feeding of breast milk and formula, defined as at least one meal of breast milk and formula daily; (3) breast milk feeding. Furthermore, the stool pattern was determined by the frequency of stool output, classified into three categories: (1) more than four times per day; (2) two to four times per day; (3) fewer than two times per day. In addition, stool color was checked for all participants. Birth data — including gender, delivery mode, birth weight, and GA— were collected from birth records.

Phototherapy was reviewed from the medical charts of nursery. The protocol of phototherapy was modified from the guideline of 2004 American Academy of Pediatrics^13^, as indicated for infants with GA 35~37 w (≥7 mg/dL at <24 h old, ≥9 mg/dL at 24–35 h old, ≥10 mg/dL at 36–47 h old, ≥12 mg/dL at 48–59 h old, ≥13 mg/dL at 60–71 h old, ≥14 mg/dL at 72–95 h old, and ≥15 mg/dL at ≥96 h old) and infants with GA >37w (≥9 mg/dL at <24 h old, ≥11 mg/dL at 24–35 h old, ≥12 mg/dL at 36–47 h old, ≥14 mg/dL at 48–59 h old, ≥15 mg/dL at 60–71 h old, ≥16 mg/dL at 72–95 h old, and ≥17 mg/dL at ≥96 h old).

### Laboratory measures

This study examined the following gene variants – including G6PD, blood type (ABO and Rh), HO-1, UGT1A1, alpha thalassemia, and SLCO1B1. We collected the umbilical cord blood samples of subjects who were born in the Chang Gung Memorial Hospital at Taipei for genomic DNA extraction. To detect the HO-1 gene containing nt 413 variant, PCR was performed using a sense primer (5′-AAG CAG TCA GCA GAG GAT TCC-3′) and an antisense primer (5′-AAC AGC TGA TGC CCA CTT TCT-3′). The examinations of G6PD, blood type, HO-1 promoter gene containing GT repeats, alpha thalassemia-1 of Southeast Asia type, SLCO1B1 gene containing nucleotide (nt) 388 variant, and UGT1A1 gene containing nt 211 variant were reported previously^4^.

### Statistical Analyses

The statistics were compiled using a commercially available program (SPSS 19.0 for Windows, SPSS Inc., Chicago, Illinois, USA). Categorical variables were analyzed using the chi-square, Fisher’s exact, or Likelihood-ratio tests when appropriate. For comparison between groups with quantitative variables, the null hypothesis that there was no difference between each group was tested by a one-way analysis of variance (ANOVA). A multivariate logistic regression model was used to estimate the risk of prolonged jaundice in relation to clinical characteristics and gene variants. Odds ratio (OR) with 95% confidence interval (CI) was expressed after adjusting for the control variables. Significance was defined as *p* < 0.05.

## Results

### Demographic information

We approached 751 parents for participation, 589 parents agreed to sign the informed consent for the collection of umbilical cord blood samples. Of the 589 infants, 509 infants were eligible for enrollment into this study at 25 to 45 days of age, including 164 with jaundice (TcB value ≥ 5 mg/dL) and 345 without jaundice (TcB value < 5 mg/dL). Their birth data are listed in Table 1. The maternal race was Asian for all infants. In addition, the birth weight was 3137 ± 357 g and 3230 ± 372 g in infants with and without jaundice, respectively. Jaundice was more common in infants with GA 35~37 weeks (52.7%) than infants with GA 38~41 weeks (29.7%). The other demographic characteristics – including sex, delivery mode, birth length, Apgar scores at 1 and 5 minutes, oxygen use, positive end-expiratory pressure (PEEP) at birth, nuchal cord, meconium stain, maternal age, and primiparity – carried no significant differences between two groups.

**Table 1:** Birth data of participants.

Birth data	Prolonged jaundice		p value
	Yes n = 164	No n = 345	
**Sex** (%)			0.368
male	80 (48.8)	183 (53.0)	
female	84 (51.2)	162 (47.0)	
**Delivery mode** (%)			0.474
vaginal	121 (73.8)	244 (70.7)	
Cesarean section	43 (26.2)	101 (29.3)	
**Gestational age** (%)			0.001
35~37 weeks	29 (17.7)	26 (7.5)	
38~41 weeks	135 (82.3)	319 (92.5)	
**Birth weight** (g)			0.762
<2500	3 (1.8)	6 (1.7)	
2500~3999	158 (96.4)	329 (95.4)	
≥4000	3 (1.8)	10 (2.9)	
**Birth length** (cm) (means ± SD)	50.4 ± 2.07	50.0 ± 2.18	0.086
**Apgar score** (means ± SD)			
at 1 min	8.96 ± 0.27	8.97 ± 0.35	0.953
at 5 min	9.98 ± 0.13	9.97 ± 0.19	0.508
**Oxygen use** (%)	0 (0)	2 (0.6)	1.000
**PEEP at birth** (%)	0 (0)	1 (0.3)	1.000
**Nuchal cord** (%)	51 (31.1)	97 (28.2)	0.489
**Meconium stain** (%)	23 (14.0)	38 (11.0)	0.329
**Maternal age** (y) (means ± SD)	33.7 ± 4.01	33.7 ± 3.91	0.994
**Primiparity** (%)	98 (59.8)	216 (62.6)	0.536

There were 44 infants with TcB ≥ 10 mg/dL (8.6%). Similarly, TcB ≥ 10 mg/dL was more common in infants with GA 35~37 weeks (20.0%) than infants with GA 38~41 weeks (7.3%) (p = 0.002).

Table 2 compares the clinical characteristics of subjects with jaundice to those without jaundice. The average age was 34.4 d and 35.7 d in infants with and without jaundice, respectively. There were significant correlations of jaundice with feeding type, stool pattern, and previous phototherapy. The incidence of jaundice was 43.1% in breastfeeding, 23.9% in combination feeding, and 0% in formula feeding. Furthermore, the incidence of jaundice was 41.5% in stool passage >4 times/d, 28.6% in stool passage 2~4 times/d, and 21.7% in stool passage <2 times/d. Overall, jaundice was more common in infants with previous phototherapy (p < 0.001), exclusive breastfeeding (p < 0.001), and stool passage >4 times/d (p < 0.001). Similarly, TcB ≥ 10 mg/dL was more common in infants with previous phototherapy (p < 0.001), exclusive breastfeeding (p < 0.001), and stool passage >4 times/d (p = 0.002). Furthermore, TcB ≥ 10 mg/dL was more common in infants with weight gain <30 g/day (p = 0.002).

**Table 2:** Clinical characteristics of participants.

Clinical characteristics	Prolonged jaundice		p value
	Yes N (%) n = 164	No N (%) n = 345	
**Feeding type**			<0.001
breast milk	113 (68.9)	149 (43.1)	
formula, combination	0 (0)	34 (9.9)	
combination	51 (31.1)	162 (47.0)	
**Stool passage** (times/d)			0.001
>4	83 (50.6)	117 (33.9)	
2~4	58 (35.4)	145 (42.0)	
<2	23 (14.0)	83 (24.1)	
**Weight gain** (g/d)			0.071
≥30	119 (72.6)	275 (79.7)	
<30	45 (27.4)	70 (20.3)	
**Previous phototherapy**			<0.001
yes	110 (67.1)	146 (42.4)	
no	54 (32.9)	198 (57.6)	

During the second visit at two months of age, no significant pathology associated jaundice was detected. In addition, no clay color stool was found during the first and second visits.

### Correlation of gene variants with prolonged jaundice

All subjects were Rh positive. The number of GT alleles in HO-1 promoter was classified into two categories: short (<24 repeats) and long (≥24 repeats) alleles^4^. Table 3 illustrates the correlation of gene variants with jaundice. Gene variant (G to A) at nt 211 of UGT1A1 was the only significant factor in relation to jaundice. The other gene variants – including G6PD, blood type, alpha thalassemia, HO-1, and SLCO1B1 – did not carry significant difference. Similarly, TcB ≥ 10 mg/dL was more common in infants with G to A at nt 211 of UGT1A1 (p < 0.001).

**Table 3:** Correlation of gene variants with prolonged jaundice by univariate analysis.

Gene variant	Prolonged jaundice		p value
	Yes N (%) n = 164	No N (%) n = 345	
ABO incompatibility	25 (15.2)	67 (19.4)	0.253
G6PD deficiency	8 (4.9)	8 (2.3)	0.122
Alpha thalassemia	10 (6.1)	20 (5.8)	0.893
UGT1A1 (G to A at nt 211)	63 (38.4)	89 (25.8)	0.004
SLCO1B1 (G to A at nt 388)	53 (32.3)	115 (33.3)	0.820
HO-1 promoter (T to A at nt 413)	61 (37.2)	134 (38.8)	0.721
short HO-1 promoter (GT)_n_ allele	71 (43.3)	136 (39.4)	0.406

### Risk assessment by multivariate logistic regression model

Table 4 shows the multivariate logistic regression analysis to assess the risk of prolonged jaundice. Possible confounders – including birth information (GA), clinical characteristics (feeding type, stool pattern, weight gain, and previous phototherapy), and genetic variants (G6PD, UGT1A1) – were included for adjustment. In this analysis, G6PD and weight gain were adjusted because we observed a trend of association. The results showed greater risks of prolonged jaundice in infants with the following four factors – GA, feeding type, previous phototherapy, and UGT1A1 gene. The most significant risk was exclusive breastfeeding, followed by previous phototherapy, GA 35~37 w, and G to A at nt211 in UGT1A1 gene. After adjusting for control variables, the other factors – including stool pattern, weight gain, and G6PD – did not carry significant risk for prolonged jaundice.

**Table 4:** Risk assessment for prolonged jaundice by multivariate logistic regression analysis.

	OR	95% CI	p value
GA 35~37 w	2.468	1.350–4.512	0.003
Exclusive breastfeeding	2.818	1.851–4.292	<0.001
Stool passage >4 times/d	1.509	1.000–2.277	0.050
Weight gain <30 g/d	1.413	0.886–2.256	0.147
Previous phototherapy	2.593	1.716–3.919	<0.001
G6PD deficiency	1.600	0.540–4.738	0.396
G to A at nt 211 in UGT1A1	1.645	1.070–2.528	0.023

The risk assessment of TcB ≥ 10 mg/dL is shown in Table 5. Adjusted factors included GA, feeding type, stool pattern, weight gain, previous phototherapy, and gene variant at nt211 of UGT1A1. The results showed greater risks of TcB ≥ 10 mg/dL in infants with exclusive breastfeeding, GA 35~37 w, previous phototherapy, G to A at nt211 in UGT1A1 gene, and weight gain < 30 g/day.

**Table 5:** Risk assessment for TcB ≥ 10 mg/dL by multivariate logistic regression analysis.

	OR	95% CI	p value
GA 35~37 w	3.591	1.565–8.240	0.003
Exclusive breastfeeding	6.234	2.555–15.21	<0.001
Stool passage >4 times/d	1.964	0.987–3.906	0.054
Weight gain <30 g/d	3.033	1.497–6.148	0.002
Previous phototherapy	2.987	1.395–6.395	0.005
G to A at nt 211 in UGT1A1	3.360	1.695–6.662	0.001

## Discussion

In this study, there is a high incidence of jaundice among infants at one month of age in Taiwan. Our study selected TcB value ≥ 5 mg/dL as an index of jaundice because visible jaundice is approximately equal to a bilirubin value of 5 mg/dL^14,15^. TcB by BiliCheck device has been a reliable measurement to assess jaundice at the range around 5 mg/dL among term and near-term infants of different races, including Asian population^16,17^. In addition to the clinical manifestations, we examined seven common gene variants involving the production and metabolism of bilirubin. Furthermore, we used a multivariate logistic regression model to control the possible confounding factors. Our results show that exclusive breastfeeding, GA 35~37 w, previous phototherapy, and gene variant of G to A at nt 211 of UGT1A1 are risk factors of jaundice for healthy infants at one month of age.

The results indicate exclusive breastfeeding as the most important factor of prolonged jaundice. In infants fed with breast milk, prolonged jaundice was very common. Our previous study demonstrated that, in breastfed infants, maternal diets are associated with prolonged jaundice^18^. The components of breast milk contribute to the development of prolonged jaundice^11^. In breastfed infants with prolonged jaundice, our clinical practice did not recommend an interruption of breastfeeding. Afterward none of these breastfed infants had pathological jaundice.

In our study, GA 35~37 w served as a risk for prolonged jaundice. As a result of immaturity in metabolism of bilirubin, preterm infants are at great risk of prolonged jaundice^19^. Our previous study enrolling 1148 healthy infants indicated that prolonged jaundice was more common in infants with GA 34~36 w than those with GA 37~42 w^18^. Genetic variant of UGT1A1 may play an important role of prolonged jaundice in preterm infants^20^.

The univariate analysis of our study showed a significant correlation of prolonged jaundice with frequent stooling. However, this correlation was not observed in the multivariate logistic regression analysis. We speculate that the stool pattern related to prolonged jaundice is caused by breastfeeding, since breastfed infants defecate more often at one month of age than those fed with formula^21^.

Our data indicated previous phototherapy as an independent risk factor of prolonged jaundice. A possible explanation is that infants with previous phototherapy might have higher bilirubin level after they were discharged from the nursery. Thus, the decline of bilirubin to normal value was supposed to be longer. In addition, we found TcB ≥ 10 mg/dL was more common in infants with weight gain <30 g/d. Inadequate feeding may cause weight loss and concurrent neonatal hyperbilirubinemia during the first few days of life^22,23^. Thus we deduce that insufficient milk feeding may contribute to the severity of prolonged jaundice. Further investigation is needed to clarify the correlation of prolonged jaundice with previous phototherapy and weight gain.

UGT1A1 is an enzyme responsible for bilirubin conjugation. A missense mutation of G to A at nt 211 in UGT1A1 is common in East Asia, including Taiwan^20,24^. In this study, G to A variant at nt 211 in UGT1A1 was associated with prolonged jaundice, which is consistent with a number of previous reports showing G to A variant at nt 211 in UGT1A1 as a risk factor of prolonged jaundice in breastfed infants^8,11^. In our study, only 6.6% of infants were not fed with breast milk at one month of age. None of infants with formula feeding had prolonged jaundice. Thus, our data cannot determine whether gene variant at nt 211 in UGT1A1 is associated with prolonged jaundice among infants fed with formula. Furthermore, we did not investigate TATA box of UGT1A1 promoter. Although gene variants of TATA box carry a significant risk of prolonged jaundice^25^, its incidence is relatively low in Taiwan^24^.

Gene variants in GT repeats or nt 413 of HO-1 promoter can modulate the production of bilirubin^26^. Nevertheless, we did not find any correlation of prolonged jaundice with HO-1 promoter genes. Our results were in contrast with a previous report by Bozkaya *et al*. showing a shorter HO-1 promoter GT repeat as a risk factor of prolonged jaundice^12^. The possible explanation is that the age of subjects was different between our and their studies. The age of subjects they included was younger and closer to the clinical manifestation of neonatal hyperbilirubinemia. Several studies have shown a shorter HO-1 promoter GT repeat was associated with neonatal hyperbilirubinemia^4,27^. Furthermore, our data showed the other common gene variants related to bilirubin production – including G6PD, blood group, and alpha thalassemia – were not relevant to prolonged jaundice. Taken together, we suggest prolonged jaundice is not associated with the production of bilirubin.

There are a couple of strengths in this study. First, our study is a prospective cohort survey, which providing more evidence-based information than case-control studies. Second, our results provide a comprehensive evidence to determine the risk of prolonged. To our knowledge, this study is the first comprehensive survey to investigate the correlation of prolonged jaundice with clinical manifestations and genetic variants among otherwise healthy infants at one month of age. However, some methodological issues should be cautiously interpreted in this study. First, we did not measure the value of conjugated bilirubin. Nevertheless, no significant pathology associated jaundice was noted during our visit at two months of age. Second, there were about 13% of missing infants in this study. We believe the bias was little because the information of missing infants was similar with that of participating infants (data not shown).

Our study systematically assessed the risk for prolonged jaundice of healthy infants at one month of age. There are some critical findings in this study. First, the polymorphism of G to A at nt 211 in UGT1A1 is the risk factor of prolonged jaundice. A high incidence of prolonged jaundice in Taiwan may be derived from gene variants of UGT1A1. Second, our epidemiological data indicate prolonged jaundice is a common condition among breast-fed infants, especially when they are preterm. Therefore, adequate support of breastfeeding is needed in this population. Third, infants with a history of phototherapy carry the risk of prolonged jaundice. In conclusion, healthy infants with GA 35~37 w, exclusive breastfeeding, previous phototherapy, or G to A at nt 211 of UGT1A1 are at greater risk of prolonged jaundice. Healthcare professionals should consider these risk factors in their assessment of prolonged jaundice. Adequate support of breastfeeding is needed for breastfed infants with prolonged jaundice.
